# Protective effects of hesperidin in gastric damage caused by experimental ischemia-reperfusion injury model in rats

**DOI:** 10.1590/acb391124

**Published:** 2024-03-11

**Authors:** Filiz Ozyigit, Ayse Nur Deger, Fatma Emel Kocak, Mehmet Fatih Ekici, Hasan Simsek, Ozlem Arık

**Affiliations:** 1 Bandirma Onyedi Eylul University – Faculty of Medicine – Department of Pharmacology – Bandirma, Turkey.; 2 Kutahya Health Sciences University – Faculty of Medicine – Department of Pathology – Kutahya, Turkey.; 3 Kutahya Health Sciences University – Faculty of Medicine – Department of Medical Biochemistry – Kutahya, Turkey.; 4 Kutahya Health Sciences University – Faculty of Medicine – Department of General Surgery – Kutahya, Turkey.; 5 Aksaray University – Faculty of Medicine – Department of Physiology – Aksaray, Turkey.; 6 Kutahya Health Sciences University – Faculty of Medicine – Department of Biostatistics – Kutahya, Turkey.

**Keywords:** Hesperidin, Ischemia, Reperfusion, Wounds and Injuries

## Abstract

**Purpose:**

This study evaluated the protective effect of hesperidin on injury induced by gastric ischemia-reperfusion.

**Methods:**

Fifty male Sprague Dawley rats (250–300 g) were divided into five groups: control (C), sham (S), ischemia (I), ischemia-reperfusion (I/R) and hesperidin + ischemia-reperfusion (Hes + I/R). Hesperidin was injected intraperitoneally at the dose of 100 mg/kg one hour before the experimental stomach ischemia-reperfusion. Celiac artery was ligated. After 45 minutes ischemia and 60 minutes reperfusion period, blood samples were obtained under anesthesia. Then, animals were sacrificed, stomach tissues were excised for biochemical, and histopathological analyses were performed. Malondialdehyde levels and superoxide dismutase, glutathione peroxidase activities and total antioxidant status (TAS), total oxidant status (TOS), protein, total thiol parameters were measured in plasma, and tissue homogenate samples. H + E, periodic acid–Schiff, hypoxia inducible factor, terminal deoxynucleotidyl transferase mediated deoxyuridine triphosphate nick end-labeling (TUNEL), and proliferating cell nuclear antigen (PCNA) for cell proliferation as immunohistochemical parameters were determined.

**Results:**

Upon biochemical and histopathological assessment, hesperidin decreased stomach tissue changes in comparison with IR group. Ischemia-reperfusion injury led to a considerably increase in malondialdehyde, protein, and TOS levels (p < 0.001) in stomach tissue. Hesperidin treatment significantly decreased malondialdehyde, protein, and TOS levels (p < 0.001). Hesperidin increased superoxide dismutase, TAS, total thiol and glutathione peroxidase activities in comparison with IR group. Hesperidin reduced damage and also increased TUNEL and PCNA immunoreactivity in stomach tissue.

**Conclusions:**

Hesperidin was able to decrease I/R injury of the stomach tissue due to inhibition of lipid peroxidation and protein oxidation, duration of antioxidant, and free radical scavenger properties. Consequently, hesperidin can provide a beneficial therapeutic choice for preventing stomach tissue ischemia-reperfusion injury in clinical application.

## Introduction

Ischemia/reperfusion (I/R) injury of the gastrointestinal tract has been linked to a high rate of morbidity and mortality in patients suffering from hemorrhagic shock, gastric ulcer bleeding, microvascular dysfunction, and gastrointestinal disorders. Due to oxidative stress, ischemia followed by reperfusion causes gastric lesions^1^.

Endothelial damage increases permeability, and cellular edema, and impairs blood flow regulation, and lysis are seen in tissues following ischemia-reperfusion (I/R)^2^. Gastric I/R leads to significant damage to the gastric mucosa^3^^,^ 4.

In the pathology of acute stomach lesions generated by I/R injury, free radicals, lipid peroxidation, and neutrophil infiltration play an important role. Increased levels of reactive oxygen species (ROS) promote a breakdown in cell membrane integrity, resulting in ulcer development^5^. Excessive formation of ROS, neutrophil adherence to endothelial cells, synthesis and release of pro-inflammatory mediators, and a change in gastric acid output are all factors in the pathophysiology of I/R-induced gastric mucosal injury. These events result in cellular damage, and increases vascular permeability, tissue necrosis, and organ failure^6^.

Free radical scavengers or antioxidants can help to protect the gastric tissue from free radical damage^7^. Hesperidin [(2*S*)-3΄,5-Dihydroxy-4ʹ-methoxy-7-[α-L-rhamnopyranosyl-(1→6)-β-D-glucopyranosyloxy]flavan-4-one], a powerful antioxidant flavanone glycoside found in the rinds of citrus fruits, has antioxidant properties similar to vitamin E^8^. In the literature, it has been reported that hesperidin can eliminate free oxygen radicals^9^. In-vitro and in-vivo studies have shown that hesperidin has antibacterial, antiviral, antihypertensive, hypolipidemic, antiulcerogenic, antineoplastic, anti-inflammatory, and antihepatotoxic effects in addition to its antioxidant action^10^^,^ 11. Since no studies on the protective effect of hesperidin in the gastric I/R injury model have been published, the purpose of this study was to investigate the protective effect and mechanism of action of hesperidin in an experimental gastric I/R injury model in rats.

## Methods

This experimental study was carried out at Kutahya Health Sciences University’s Experimental Animal Center in Kutahya, Turkey, after receiving approval from the Animal Experiments Local Ethics Committee (Decision Number: 2014.08.08). Rats were purchased from Kutahya Health Sciences University’s Experimental Animal Center. All experiments were carried out in accordance with the Guide for the Care and Use of Laboratory Animals published by the Institute of Laboratory Animal Resources Commission on Life Sciences National Research Council^12^.

### Materials

Hesperidin (Hesperetin 7-rhamnoglucoside; Cirantin; Hesperetin-7-rutinoside, C_28_H_34_O_15_, MW: 610.56, purity ≥ 95%, CAS Number: 520-26-3) was purchased from Santa Cruz Biotechnology, Inc. (Santa Cruz, CA, United States of America). The compound was dissolved in a saline solution containing 0.5% Tween-80. Hesperidin was administered intraperitoneally (i.p.) at doses of 100 mg/kg. Drug administration was performed 60 minutes before the surgical procedure. Hesperidin doses were preferred based on previous experiments^13^.

### Animal model

Adult male Sprague Dawley rats weighing 250 to 300 g were used. Rats were taken to the laboratory one week prior to starting work to ensure compliance with the medication. They were fed pellet feed and drank water during the experiment. All animals were housed 12 hours in darkness, 12 hours in light, and at optimal room temperature. They were placed into five transparent polycarbonate cages in groups of five. The rats were randomly divided into five groups (n = 10 each group) as follows:

- Group 1: control (C) group, in which the stomach was removed without any surgical treatment protocol and I/R;
- Group 2: sham (S) group, in which the rats were subjected to identical surgical procedure, but no I/R. Sisty minutes before the laparotomy, 1 mL of saline was given;
- Group 3: gastric ischemia injury (I) group;
- Group 4: gastric ischemia / reperfusion injury (I/R) group;
- Group 5: hesperidin + I/R (H + I/R) group, in which the rats were given 100 mg/kg hesperidin i.p. 60 minutes before the surgical procedure.

### Preparation of tissue homogenates

For biochemical analysis, tissue samples were mixed with a cold working solution (50 mM phosphate buffer, pH 7.40), and homogenized with a mechanical homogenizer (Analytik Jena speedmill plus, Jena, Germany). The mixtures were then centrifuged at 10,000 × g for 15 min at 4°C, and the supernatants were preserved for biochemical analysis by storing on ice.

### Surgical procedures

The animals were weighed and then anesthetized with i.p. injections of 70-mg/kg ketamine (Ketalar; Pfizer, Istanbul, Turkey) and 10-mg/kg xylazine hydrochloride (Rompun; Bayer, Istanbul, Turkey). During the surgery, the body temperature was maintained at 37°C with a homeothermic dissection table. The I/R injury model was carried out as previously described^14^. The surgical procedure was performed after the surgical incision site was cut, and the skin was cleaned with povidone iodine. Experimental animals underwent laparotomy with an anterior medial incision of the anterior abdominal wall under anesthesia. The celiac artery was clamped with an atraumatic vascular clamp (Vascu Stop, Bulldog Clamp) for 45 minutes. Ischemia was evaluated by the absence of pulses or the pale color of the stomach. Following ischemia, the clamp was removed, and a 60-minute reperfusion was performed. The start of the beats and the return of the red color were accepted as signs of stomach reperfusion.

### Laboratory analysis

#### Sample collection

After 60 min of reperfusion, the experiment was terminated. The animals were sacrificed under anesthesia, and the stomach was carefully excised. The gastric tissue samples were rinsed with a cold heparinized saline solution to remove any red blood cells or clots. A portion of the stomach samples were fixed with 10% buffered formalin for histopathological and immunohistochemical analyses. Another portion of gastric tissue samples were placed into Eppendorf tubes and immediately stored at -80°C until biochemical analysis.

#### Preparation of tissue homogenates

For biochemical analysis, tissue samples were mixed with a cold working solution (50 mM phosphate buffer, pH 7.40), and homogenized with a mechanical homogenizer (Analytik Jena speedmill plus, Jena, Germany). The mixtures were then centrifuged at 10,000 × g for 15 min at 4°C, and the supernatants were preserved for biochemical analysis by storing on ice.

#### Measurement of tissue total antioxidant status, total oxidant status, and total thiol levels

Tissue total antioxidant status (TAS), total oxidant status (TOS), and total thiol (TT) levels were measured on the Beckman Coulter AU680 analyzer (Beckman Coulter, Miami, FL, United States of America) using commercial reagents (Rel Assay Diagnostic, Gaziantep, Turkey) based on novel automated measurement methods developed by Erel^15^^–^ 17. TAS levels were expressed as mmol Trolox Eq/mg protein. TOS levels were expressed as mmol Trolox Eq/mg protein. TOS levels were expressed as μmol H_2_O_2_ Eq/mg protein. TT levels were expressed as μmol/mg protein.

#### Calculation of the oxidative stress index

The percent ratio of TOS to TAS was accepted as the oxidative stress index (OSI), an indicator of the degree of oxidative stress. To calculate OSI, the unit of TAS, mmol of Trolox equivalent per mg of protein, was converted to mol of Trolox equivalent per mg of protein, and the Eq. 1^18^ was used:


$$OSI=\left[\left(TOS,molH_{2}O_{2}Eq/mg\text{protein}\right)/(TAS,\text{mol Trolox Eq/mg protein})100\right]$$ (1)

#### Measurement of tissue superoxide dismutase and glutathione peroxidase activities

Tissue superoxide dismutase (SOD) activities were measured on the Beckman Coulter AU680 analyzer (Beckman Coulter, Miami, FL, United States of America) using the Ransod kit (Randox Laboratories Ltd., Crumlin, UK). SOD activities were expressed as U/mg protein. Tissue glutathione peroxidase (GSH-PX) activities were measured on the Beckman Coulter AU680 analyzer (Beckman Coulter, Miami, FL, United States of America) using the Ransel kit (Randox Laboratories Ltd., Crumlin, United Kingdom). GSH-PX activities were expressed as U/mg protein.

#### Measurement of tissue malondialdehyde (MDA) levels

Tissue malondialdehyde (MDA) levels were measured based on the thiobarbituric acid reactive substances method using commercial enzyme-linked immunosorbent assay (ELISA) kits (Cayman Inc., Ann Arbor, MI, United States of America) on a microplate reader (BMG Labtech Spectrostar Nano, GmbH, Ortenberg, Germany). MDA levels were expressed as μM/mg protein.

#### Measurement of tissue protein level

Tissue protein levels were measured based on the Bradford method on the Beckman Coulter AU680 analyzer (Beckman Coulter, Miami, FL, United States of America)^19^.

### Histopathologic examinations

Gastric mucosa samples were fixed in 10% formaldehyde solution, embedded in paraffin, and 4-micron thick sections were prepared. Hematoxylin-eosin (H&E) staining was applied to the sections after deparaffinization and graded hydration and studied under a light microscope (Olympus BX41; Tokyo, Japan) by a pathologist who was blinded to the study groups. The scales used by Zhang et al. were used to evaluate H&E stained sections^20^. Mucosal epithelium was graded from 0 to 3 according to the injury (including point erosion, ulcer, and point hemorrhage):

1. 0: no ulcer and no erosion;
2. 1: erosion in the length of ≤ 1 mm;
3. 2: > 1-mm and ≤ 2-mm long erosion;
4. 3: > 2-mm and ≤ 3-mm long erosion.

If the width of the lesion were > 1 mm, the scale was folded to 2. Muscle loss in stomach epithelium with periodic acid–Schiff (PAS) was graded from 0 to 3^20^^,^ 21.

### Immunohistochemical examinations

Hypoxia inducible factor-1α (HIF-1α) and proliferating cell nuclear antigen (PCNA) were examined immunohistochemically. Immunohistochemical staining was performed using commercially available kits (HIF-1α, Santa Cruz sc-10790; PCNA abcam ab18197) according to the manufacturer’s instructions. Gastric tissue was paraffinized and rehydrated. Four–five-micron thick sections were prepared for immunohistochemical evaluation with PCNA and HIF-1α. Endogenous peroxidase activity was blocked with 0.5% hydrogen peroxide in methanol. Primer antibodies to PCNA and HIF-1α were applied for 60 minutes. Peroxidase activity was elevated with DAB. Hematoxylin was applied for contour staining. Positive-responding cells were counted at 400x magnification. All the histomorphological and immunohistochemical evaluations were performed using the Olympus CX 41 light microscope. In all, seven randomly selected counties were included in the immunohistochemical assessment. The percentage of cells giving positive reactions was calculated.

### In-situ detection of apoptosis

We used an in-situ terminal deoxynucleotidyl transferase mediated deoxyuridine triphosphate nick end-labeling (TUNEL) assay to determine the degree of stomach apoptosis. For the TUNEL, coloring Roche 11684817910 kit was used. The sections prepared from paraffin embedded tissues were deparaffinized with xylene. It was then passed through 95% ethanol, 85% ethanol, 75% ethanol, and 70% ethanol. The sections were washed with phosphate buffered saline (PBS) for 5 minutes. Proteinase K was incubated at room temperature for 20 minutes. After washed twice with PBS, the tissues were subjected to TUNEL at 37°C for 60 minutes. TUNEL positive cells were evaluated under 400x magnification under a light microscope. Apoptotic cells were counted^22^.

### Statistical analysis

The data were analyzed by Statistical Package for the Social Science version 16.0 (SPSS Co., Chicago, IL, United States of America) for Windows. Numerical data were tested for normality using the Shapiro-Wilk’s test. While normally distributed data were expressed as mean ± standard deviation (SD), not normally distributed data were expressed as the median and interquartile range (IQRs). The comparisons of numerical variables among groups were tested using analysis of variance (ANOVA) and Kruskal-Wallis’ tests according to the distribution of data. Homogeneity test of variances of variables was performed with the Levene’s statistic to select the post-hoc test. The multiple comparisons of examined variables were tested using the Bonferroni multiple comparisons post-hoc test for variables that passed the homogeneity of variances test and using the Dunnett T3 multiple comparisons post-hoc test for variables that no passed homogeneity of variances test. Statistical significance was established at the level of P < 0.05.

## Results

### Tissue total antioxidant status, total oxidant status, oxidative stress index, and thiol levels

Differences in tissue TAS, TOS, OSI, and TT levels between assay groups are represented in Table 1. Significant difference was observed among groups for TAS, TOS, OSI, and TT values (P < 0.0001, P < 0.0001, P < 0.0001, P < 0.0001, respectively). I/R caused significant increases in TOS and OSI levels (P < 0.05) and significant decreases in TAS and TT levels (P < 0.05) in comparison with those caused by the control and sham groups. Hesperidin decreased TOS and OSI levels and increased TAS and TT levels compared to I/R group (P < 0.05).

**Table 1:** Differences in tissue TAS, TOS, OSI, TT, MDA levels, and tissue SOD and GSH- PX activities between assay groups.

Parameters group 1 (mean ± SD) (Control) (n = 10)	Group 2 (Sham)(n = 10)	Group 3 (Ischemia) (n = 10)	Group 4 (I/R)(n = 10)	Group 5 (H + IR) (n = 10)	P-value
TAS(mmol Trolox 0.11 ± 0.013 Eq/mg protein)	0.11 ± 0.019	0.072 ± 0.013^ab^	0.042 ± 0.01^abc^	0.076 ± 0.015^abd^	< 0.0001
TOS 0.022 ± 0.015(μmol H_2_O_2_ Eq/mg protein)	0.027 ± 0.013	0.058 ± 0.014^ab^	0.078 ± 0.015^ab^	0.049 ± 0.019^abd^	< 0.0001
OSI (Arbitrary 0.02 ± 0.013 Unit)	0.024 ± 0.01	0.083 ± 0.021^ab^	0.19 ± 0.031^abc^	0.067 ± 0.021^abd^	< 0.0001
SOD (U/mg 1.39 ± 0.22 protein)	1.25 ± 0.15	0.82 ± 0.10^ab^	0.53 ± 0.14^abc^	1.07 ± 0.26^acd^	< 0.0001
GSH-PX (U/mg 7.69 ± 1.73 protein)	7.34 ± 1.34	4.02 ± 1.12^ab^	2.60 ± 0.65^abc^	4.58 ± 1.42^abd^	< 0.0001
MDA (μM/mg 0.23 ± 0.06 protein)	0.24 ± 0.08	0.70 ± 0.20^ab^	0.76 ± 0.14^ab^	0.48 ± 0.13^abcd^	< 0.0001
TT (μmol/mg 22.5 ± 3.34)	21.5 ± 4.07	10.31 ± 2.33^ab^	7.08 ± 1.15^abc^	11.46 ± 2.83^abd^	< 0.0001

IR: ischemia/reperfusion; H: hesperidin; TAS: total antioxidant status; TOS: total oxidant status; OSI: oxidative stress index; SOD: superoxide dismutase; GSH-PX: glutathione peroxidase; MDA: malondialdehyde; TT: total thiol. Results are expressed as the mean ± standard deviation (SD). Since all data were normally distributed, the comparisons of variables among groups were analyzed with the one-way analysis of variance; p-value shows the differences among all groups. Since all data were passed the homogeneity of variances test, the multiple comparisons between two groups were analyzed with the Bonferroni-post-hoc test; (a) compared to control group; (b) compared to sham group; (c) compared to ischemia group, (d) compared to I/R group. Source: Elaborated by the authors.

### Tissue superoxide dismutase and glutathione peroxidase activities

Differences in tissue SOD and GSH-PX activities between assay groups are represented in Table 1. Significant difference was observed among groups for SOD and GSH-PX activities (P < 0.0001, P < 0.0001, respectively). SOD and GSH-PX activities in the I/R group were significantly lower compared to the control and sham groups (P < 0.05). Hesperidin increased SOD and GSH-PX activities when compared to the I/R group (P < 0.05).

### Tissue malondialdehyde levels

Differences in tissue MDA levels between assay groups are represented in Table 1. Significant difference was observed among groups for MDA levels (P < 0.0001). The group subjected to IR showed significantly increased in MDA levels compared to those of the control and sham groups (P < 0.05). Hesperidin decreased MDA levels compared to the I/R group (P < 0.05).

### Histopathological and immunohistopathologic results

Comparisons of scored histopathological and immunohistochemical values in gastric tissue samples among assay groups are represented in Table 2. In the histopathological examinations, our results demonstrated that I/R injury caused a marked increase in the total histopathological gastric damage score compared with the control and sham groups (P < 0.05, P < 0.05, respectively). A dose of 100 mg/kg of hesperidin significantly reduced the gastric damage scores compared to the I/R group (P < 0.05).

**Table 2:** Comparisons of scored histopathological and immunohistochemical values in gastric tissue samples among assay groups.

Parameters (mean ± SD)		Group 1	Group 2	Group 3	Group 4	Group 5	P-value
		(Control)	(Sham)	(Ischemia)	(I/R)	(H + IR)	
Gastric damage		(n = 10) 0.0 (0.0–0.0)	(n = 10) 0.0 (0.0–0.0)	(n = 10) 2.0 (1.0–2.0)^ab^	(n = 10) 2.0 (2.0–2.0)^ab^	(n = 10) 1.0 (1.0–1.0)^abcd^	< 0.0001
(H&E)							
	Apoptotic index	2.2 ± 1.2	2.3 ± 1.0	11.1 ± 3.5^ab^	14.8 ± 3.9^ab^	6.1 ± 1.2^abcd^	< 0.0001
	HIF-1α	8.8 ± 3.4	7.9 ± 4.2	14.8 ± 5.8^b^	16.9 ± 6.3^ab^	9.2 ±. 5.6^d^	< 0.0001
	PCNA	41.3 ± 14.2	33.0 ± 8.1	19.9 ± 2.1^ab^	15.7 ± 5.3^ab^	31.0 ± 5.8^cd^	< 0.0001
	PAS	0.0 (0.0–0.0)	0.0 (0.0–0.0)	2.0 (1.0–2.0)^ab^	2.0 (2.0–2.3)^ab^	1.0 (1.0–1.3)^abd^	< 0.0001

I/R: ischemia/reperfusion; H: hesperidin; HIF-1α: hypoxia-inducible factor-1α; PCNA: proliferating cell nuclear antigen; PAS: periodic acid-Schiff; H&E: hematoxylin and eosin. Normally distributed data were expressed as mean ± standard deviation (SD), and not normally distributed data were expressed as the median and interquartile range (IQRs). The comparisons of variables among groups were tested using analysis of variance and Kruskal-Wallis’ tests according to the distribution of data; p-value shows the differences among all groups. The multiple comparisons between two groups were analyzed with the Bonferroni post-hoc test for HIF-1α that passed the homogeneity of variances test and with the Dunnett T3 post-hoc test for the other variables that no passed homogeneity of variances test; (a) compared to control group; (b) compared to sham group; (c) compared to ischemia group, (d) compared to I/R group. Source: Elaborated by the authors.

**Figure 1:**
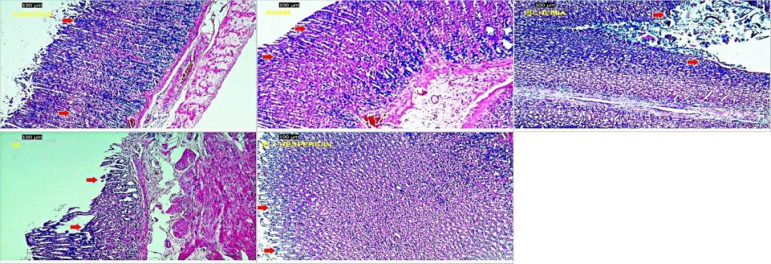
Histopathological examination of gastric tissues in assay groups, hematoxylin and eosin (H&E) × 100. The control and sham groups contained regular stomach mucosa, epithelium. Ischemia group ulcer surface epithelium, grade 2. ischemia/reperfusion group, ulcer surface epithelium grade 2. Hesperidin + ischemia/reperfusion group, improvement in epithelial ulceration, ulcerated epithelium grade 1.

Source: Elaborated by the authors.

**Figure 2:**
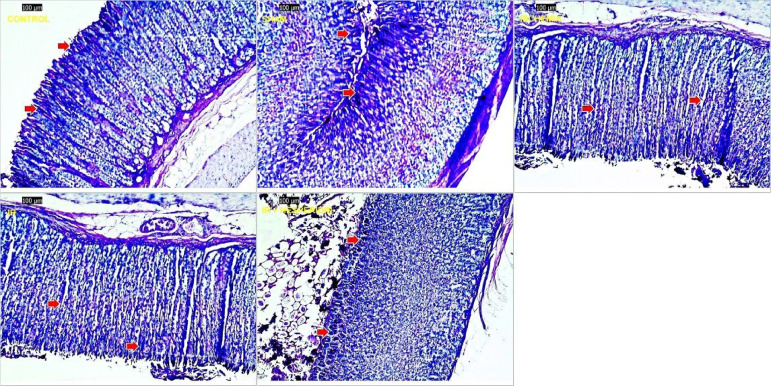
Histopathological examination of stomach tissues in assay groups, periodic acid–Schiff (PAS) × 100. The control and sham groups contained regular stomach mucosa, mucin-containing epithelium. Ischemia group, decreased mucin grade 2 in epithelium. Ischemia/reperfusion group, decreased mucin grade 3 in epithelium. Hesperidin + ischemia/reperfusion group, improvement of epithelial mucin loss, cells containing mucin (PAS indicated by a red arrow).

Source: Elaborated by the authors.

**Figure 3:**
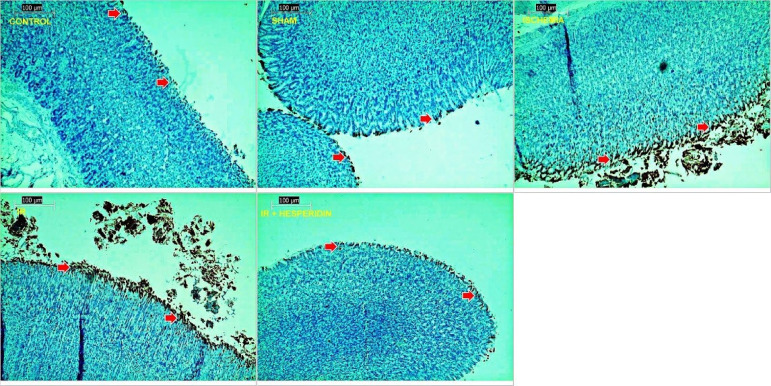
Examination of apoptosis in stomach tissues in assay groups, terminal deoxynucleotidyl transferase mediated deoxyuridine triphosphate nick end-labeling (TUNEL) × 400. The number of TUNEL positive cells per 100 was calculated from randomly selected fields. The apoptotic index was calculated as the percentage of apoptotic (TUNEL-positive stained) cells. The control and sham groups, TUNEL positive few cells, ischemia group, positive reaction with more number of cells, ischemia/reperfusion (I/R) group, positive reaction with more number of cells with more number of cells, I/R-group, positive reaction with more number of cells with TUNEL, I/R injury caused a significant increase in the apoptotic index, hesperidin + I/R group, positive reaction with fewer cells compared with ischemia and I/R groups with TUNEL. Hesperidin significantly decreased the apoptotic index. TUNEL-positive stained indicated by a red arrow.

Source: Elaborated by the authors.

Compared with the control and sham group, significant surface ulcers were observed in the I/R group (grade 2). However, improvement in the epithelial ulceration was seen in the H + I/R group (grade 1) (Figs. 1 and 2). When apoptosis in the gastric tissue was examined using the TUNEL method, we observed that I/R injury caused a significant increase in the apoptotic index compared with the control and sham groups (P < 0.05, P < 0.05, respectively). A dose of 100 mg/kg of hesperidin significantly reduced the apoptotic index compared with the I/R group (P < 0.05) (Fig. 3). The effect of hesperidin on cell proliferation was examined by PCNA immunostaining. Study results demonstrated that I/R injury caused a significant decrease in the number of PCNA positive cells compared with the control and sham group (P < 0.05, P < 0.05, respectively).

**Figure 4:**
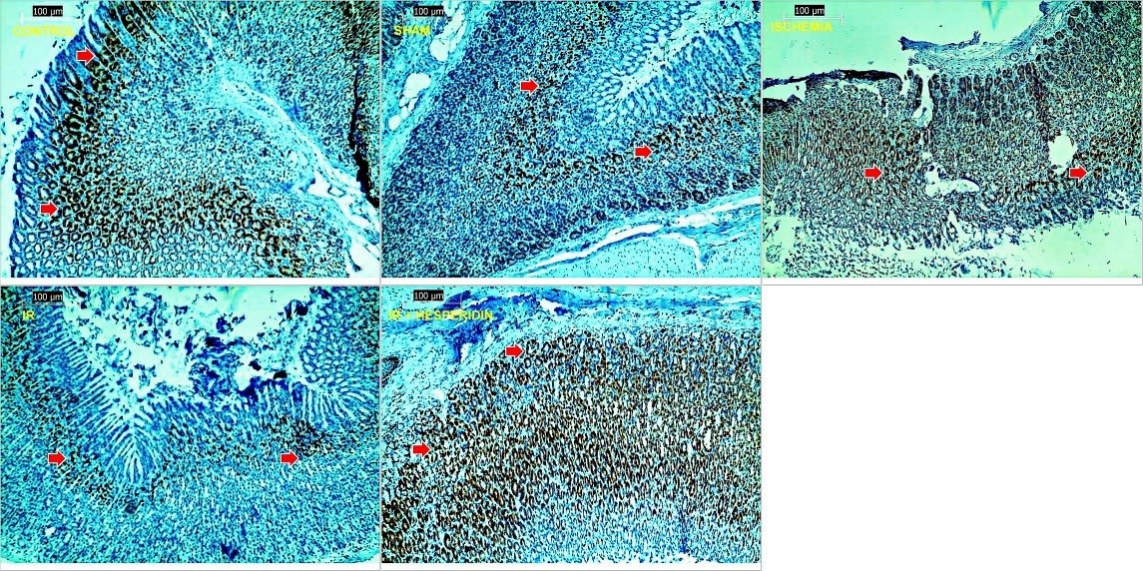
Examination of proliferating cell nuclear antigen (PCNA) expressions in stomach tissues in assay groups, PCNA × 400. The control and sham groups diffuse positive reaction with PCNA, ischemia group, PCNA positive reaction in a small number of cells, ischemia/reperfusion group, positive reaction with PCNA in a small number of cells. The percentage of PCNA positive cells was evaluated. Ischemia/reperfusion injury caused a significant decreased in the number of PCNA positive cells, hesperidin + ischemia/reperfusion group, positive reaction with PCNA in a larger number of cells compared with ischemia and ischemia/reperfusion groups. Hesperidin increased the number of PCNA positive cells compared with ischemia/reperfusion group. PCNA is indicated by a red arrow.

Source: Elaborated by the authors.

**Figure 5:**
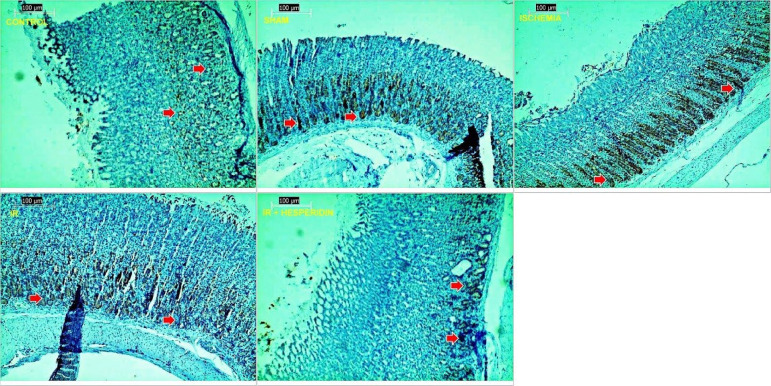
Examination of hypoxia inducible factor-1α (HIF-1α) expressions in stomach tissues in assay groups, HIF-1α × 400. The control group contained a small number of positive cells, sham group, a few cells positive, ischemia group, positive reaction with more number of cells, ischemia/reperfusion group, positive reaction in more cells. When compared to the control and sham groups, ischemia/reperfusion damage induced a significant increase in the amount of HIF-1α positive cells, but hesperidin reduced the amount of HIF-1α positive cells. HIF-1α is indicated by a red arrow.

Source: Elaborated by the authors.

However, hesperidin increased the number of PCNA positive cells compared to I/R group (P < 0.05) (Fig. 4). HIF-1α was examined using immunohistochemical staining. Study results demonstrated that I/R injury caused a significant increase in HIF-1α compared with the control and sham group (P < 0.05, P < 0.05, respectively). Hesperidin pre-treatment reduced the number of HIF-1α positive cells compared to I/R group (P < 0.05) (Fig. 5).

## Discussion

Gastrointestinal mucosa is one of the most sensitive tissues to the ischemia. Cytotoxic effects of free oxygen radicals occurring after I/R and lipid peroxidation (LPO) play an important role in the formation of gastric mucosal lesions^23^. Increased free radicals and acidity of the I/R result in erosion and ulceration of the gastric mucosa. As a result, secretion and absorption functions deteriorate, and mucosal necrosis develops^24^^,^ 25. Gastric I/R is a complicated situation that limited information can be reached even though there are so many investigations on the damage^26^^,^ 27. Decreased or cut off blood flow in the stomach and reperfusion that occurs after it causes tissue damage at various grades. The I/R model can be created experimentally, as well as in many other organs. I/R injury increases free radical formation in the gastric mucosa, leading to LPO.

However, many studies have shown that antioxidants and free radical scavengers have gastroprotective effects by inhibiting LPO. Many antioxidant substances have the gastroprotective effect in experimental models of stomach I/R injury, which has recently been confirmed by different studies, even though there is no study of this model with hesperidin^28^^,^ 29.

Degradation of oxidant and antioxidant balance in tissues is caused by oxidative stress. This causes serious damage to the gastric mucosa and negatively affects normal functions during I/R. Oxidative stress causes an increase in the products of LPO in the cellular membranes^30^. Our results indicate that the mechanisms underlying the protective actions of hesperidin against gastric I/R may be related to its capability to decrease the MDA levels. In this study, we observed an increase in MDA levels in the I/R group compared to the control and sham groups. Administration of hesperidin prior to I/R provided a protective effect against gastric I/R injury by reducing MDA levels. These results were consistent with a previous study in which 100 mg/kg hesperidin was administered i.p. Consequently, hesperidin pretreatment may demonstrate protective effects against gastric I/R injury by reducing LPO^31^.

Increased free oxygen radicals (SOR) plays an important role in functional disorders in gastric I/R injury. Although the cells can protect themselves against oxidative damage, the antioxidant system is deficient in I/R damage. Endogenous antioxidant enzymes such as SOD and GPx in organism play an important role in reducing SOR’s harmful effects. However, the levels of these enzymes fall during reperfusion in studies on I/R models. This is probably the result of an increase in free radicals^32^^–^ 34. During ischemia, the acceleration in the inactivation of enzymes such as SOD, GPX is observed due to the oxidants in the ischemic tissue. During the reperfusion process, cells are more susceptible to the effects of oxygen radicals. The improvement of I/R damage depends on the ability of the gastric mucosa to renew itself^35^. In this study, we found a significant decrease in antioxidant enzyme activities (SOD, GPx) in the I/R group compared with control and sham groups. In addition, we found that hesperidin at doses of 100 mg/kg appeared to increase antioxidant enzyme activities.

Our results were consistent with previous studies in which a 100 mg/kg dose of hesperidin was given^32^^–^ 34 ^,^ 36. The protective effect of hesperidin against gastric I/R injury may have been achieved through the antioxidant defense system. After 45 minutes of ischemia and 60 minutes of reperfusion, we observed that TOS and OSI levels, which are indicators of damage caused by free oxygen radicals, increased in the I/R group. Hesperidin pre-treatment reversed all these results.

Hesperidin has been reported to play a protective role in many experimental models with different doses, routes of administration, and administration times. Korthuis and Gute reported that damage to their work by inducing intestinal I/R injury in mice can be greatly reduced by hesperidin^37^. Dorkina demonstrated the hepatoprotective effect of hesperidin at a 100-mg/kg dose in their study of acute hepatitis by giving CCL4 to guinea pigs^38^.

HIF-1 is one of the regulators which play a key role in ischemia. It consists of two subunits, α and β. The α subunit is closely related to cellular oxygen sensitivity. HIF-1α increases in sensitivity to hypoxia during ischemia^39^. In our study, the percentages of HIF-1α-positive cells were significantly increased in the I/R group when compared to the control and sham groups. The ratio of HIF-lα positive cells was lower in the hesperidin-treated group than in the I/R group. Therefore, hesperidin may be protective against gastric I/R injury by lowering HIF-1α protein levels.

In Qiao et al.’s study, although the gastric mucosa is mildly affected during ischemia, it has been found that more severe damage occurs, especially during the first hour of reperfusion, and that the ability of the stomach mucosa to repair itself was extremely high and that it was completed on the third day of complete repair. They argued that this was achieved by inhibiting gastric mucosal apoptosis and increasing the number of proliferative cells^40^.

There are limited studies on gastric mucosal apoptosis and proliferation. Some studies contain different results. In the stomach mucosa, apoptotic cells, and proliferative cells are pointed out together^41^.

Stomach I/R damage is associated with increased gastric mucosal injury, apoptosis, and decreased gastric mucosal cell proliferation. PCNA is present at various concentrations within the cell throughout the cell cycle. PCNA is an accessory protein that is secreted during the maximum S phase of cell cycle. The effect of hesperidin on cell proliferation was confirmed by histopathological analysis and PCNA immunostaining^42^.

Hesperidin at the dose of 100 mg/kg caused an increase in cell proliferation. The TUNEL method was used to determine the apoptotic cells in the tissues. According to the TUNEL method results, the number of cells stained positive in the ischemia and I/R groups was higher than the control group, and this difference was statistically significant. These findings are similar to the protective effect of hesperidin in experimental I/R models. TUNEL for apoptosis and PCNA for cell proliferation in our study were similar to these findings^43^^,^ 44. Hesperidin caused a decrease in apoptosis and an increase in cell proliferation. Our study findings suggest that oxidative stress plays an important role in gastric I/R injury and that hesperidin pre-treatment may be useful against this damage.

## Conclusion

This study suggests that hesperidin may have protective effects in gastric I/R injury. The mechanism underlying the protective effects of hesperidin in preventing damage due to gastric I/R injury is related to the ability to reduce lipid peroxidation, increase endogenous antioxidant enzyme activities, reduce apoptosis, and increase proliferation.

## Data Availability

All data sets were generated or analyzed in the current study.
